# Undefined and fatal meningoenchephalitis in a patient with combined immune deficiency: a case report

**DOI:** 10.1186/1939-4551-8-S1-A165

**Published:** 2015-04-08

**Authors:** Karla Boufleur, Myrthes Toledo Barros, Myrthes Toledo Barros, Leonardo Mendonça, Fabiana Mascarenhas, Pablo Torres, Cristina Kokron, Ana Karolina Barreto De Oliveira, Luiz Augusto Marcondes, Nathalia Siqueira Robert De Castro, Jorge Kalil

**Affiliations:** 1 Hcfmusp, Brazil; 2 Hospital Das Clínicas - Faculdade De Medicina – USP, Brazil; 3 University of São Paulo, Brazil; 4 Laboratory of Clinical Immunology and Allergy (LIM-60), University of São Paulo Medical School, Brazil

## Background

Encephalitis is defined by the presence of brain inflammation associated with clinical evidence of neurological dysfunction. It can be due to infection (most common cause is viruses) or not (like post vaccine or auto immune). Primary immunodeficiency is defined as a genetic basis that leads any alteration in immune system (innate or adaptative) predisposing to infections, auto immunity and malignancies. Combined immunodefiency,alteration in T and B cell function, are potentially fatal disease.

## Methods

Review of clinical data from eletronic records.The objective of this paper is relate a case of a patient with encephalitis and our difficulty in establishing the etiology

## Results

L.S.N., 25 years old female patient followed by combined immunodeficiency presented with acute arthritis, nodosum erythema and acute seizures. The liquor analysis evidenced lymphocitic pleocitosis with elevated protein levels. No alterations were evidenciated in brain CT .The patient underwent an uncontrolled epileptic status that required intubation,sedation, broad-spectrum antibiotics, anti-fungal, anti-viral, tuberculostatic agents and corticosteroids. Despite all clinical investiment the patient had a fatal cardiac arrest.

## Conclusions

Establish specific sorological etiology of clinical manifestations in primary immunodeficiencies is essential but a chalenge in clinical practice. New techniques in the diagnosis of infections and autoimmune conditions are necessary.

## Consent

Written informed consent was obtained from the patient for publication of this abstract and any accompanying images. A copy of the written consent is available for review by the Editor of this journal.

